# Serum concentration of alpha-1 antitrypsin is significantly higher in colorectal cancer patients than in healthy controls

**DOI:** 10.1186/1471-2407-14-355

**Published:** 2014-05-21

**Authors:** Sergio Pérez-Holanda, Ignacio Blanco, Manuel Menéndez, Luis Rodrigo

**Affiliations:** 1 General Surgery Department, Hospital Valle del Nalón, 33920 Langreo, Principality of Asturias, Spain; 2 Board of Directors of the Alpha1-Antitrypsin Deficiency Spanish Registry, Spanish Society of Pneumology (SEPAR), Spanish Lung Foundation Breathe, Provenza, 108, 08029 Barcelona, Spain; 3 Clinical Biochemistry Department, Instituto Nacional de Silicosis, Hospital Universitario Central de Asturias, 33006 Oviedo, Principality of Asturias, Spain; 4 Department of Gastroenterology, Hospital Universitario Central de Asturias, 33006 Oviedo, Principality of Asturias, Spain

**Keywords:** Alpha-1 antitrypsin, Serum concentration, Gene frequency, Colorectal cancer

## Abstract

**Background:**

The association between alpha-1 antitrypsin (AAT) deficiency and colorectal cancer (CRC) is currently controversial. The present study compares AAT serum concentrations and gene frequencies between a group of CRC patients and a control group of healthy unrelated people (HUP).

**Methods:**

267 CRC subjects (63% males, 72 ± 10 years old) were enlisted from a Hospital Clinic setting in Asturias, Spain. The HUP group comprised 327 subjects (67% males, mean age 70 ± 7.5 years old) from the same geographical region. Outcome measures were AAT serum concentrations measured by nephelometry, and AAT phenotyping characterization by isoelectric focusing.

**Results:**

Significantly higher serum concentrations were found among CRC (208 ± 60) than in HUP individuals (144 ± 20.5) (p = 0.0001). No differences were found in the phenotypic distribution of the Pi*S and Pi*Z allelic frequencies (p = 0.639), although the frequency of Pi*Z was higher in CRC (21%) than in HUP subjects (15%).

**Conclusions:**

The only statistically significant finding in this study was the markedly higher AAT serum concentrations found in CRC subjects compared with HUP controls, irrespective of whether their Pi* phenotype was normal (Pi*MM) or deficient (Pi*MS, Pi*MZ and Pi*SZ). Although there was a trend towards the more deficient Pi* phenotype the more advanced the tumor, the results were inconclusive due to the small sample size. Consequently, more powerful studies are needed to reach firmer conclusions on this matter.

## Background

Human alpha-1 antitrypsin (AAT), also known as alpha1 proteinase inhibitor (α1-Pi) and SERPINA1 (Serine Protease Inhibitor, group A, member 1), is a circulating glycoprotein whose main function is to inhibit neutrophil elastase and other serine proteases in blood and tissues. The AAT gene has two alleles, which are transmitted from parents to their children by autosomal co-dominant Mendelian inheritance. Normal alleles, present in 85-90% of individuals, are denominated Pi (protease inhibitor) M. Thus, a normal individual has a Pi*MM genotype. The most prevalent deficiency alleles are denominated S and Z, and their prevalence in Caucasian populations ranges from 5-10% and 1-3%, respectively. Consequently, the vast majority of genotypes result from combinations of Pi*M, Pi*S and Pi*Z. The normal genotype, Pi*MM, is present in about of 85-95% of people and fully expresses AAT; Pi*MS, Pi*SS, Pi*MZ, Pi*SZ and Pi*ZZ are deficiency genotypes that are present in the other 5-15%, expressing approximately 80, 60, 55, 40 and 15% of AAT, respectively [1].

Severe AAT deficiency, defined as an AAT serum level less than 35% of the mean expected value, 50 mg/dL (measured by nephelometry), 11 μM, or 80 mg/dL (measured by radial immunodifusion, although this is now an obsolete technique), is usually associated with Pi*ZZ genotypes, and less frequently with combinations of Z, S, and about 45 “rare” or null alleles. Both Pi*S and Pi*Z, and the rare deficiency alleles MMalton, MDuarte, and SIiyama produce misfolded proteins that are retained in polymer-forming hepatocytes. These can cause not only cell stress and liver damage, but also, as a result of polymerization and retention in hepatocytes, blood and tissue concentrations of AAT that are too low to provide sufficient protection for tissues against the action of proteinases [2].

AAT deficiency is a hereditary condition that typically predisposes to premature onset of chronic obstructive pulmonary disease (COPD), liver cirrhosis, relapsing panniculitis, systemic vasculitis, and possibly a range of inflammatory and neoplastic diseases [1,3]. In addition, several clinical studies have shown that subjects with AAT deficiency have an increased risk of developing malignancies, including hepatocellular carcinomas [4,5], lung cancer [6-9], neoplasms of the urinary bladder [10] and gallbladder [11], malignant lymphomas [12], and colon cancers [13,14].

The aim of the present study was to investigate whether AAT deficiency was more common in patients with CRC than in healthy subjects from Asturias, a northern (Cantabrian) coastal region of Spain, with one of the highest prevalences of AAT deficiency in Europe [15,16], and a high incidence of CRC [17].

## Methods

### Type of study

This is a population-based genetic project that was designed as a case-control study comparing CRC patients with a control group of healthy unrelated people (HUP) from the central region of Asturias, which has an area of 646 km^2^ and a population of 78,315 inhabitants, almost all of whom are Caucasian. The population has not changed significantly in recent last years, and has been little influenced by interbreeding, devastating natural disasters, wars, epidemics, or migration. This means that the popoulation may be assumed to be in Hardy-Weinberg equilibrium, enabling us to estimate the prevalence of the different phenotypes of AAT in the population.

### Ethics

The project was approved by the Valle del Nalón Hospital Clinical Research Committee (Decision 1/2008). The study was carried out according to the Good Clinical Practice Guidelines of the modified Helsinki declaration. Specific signed informed consent was obtained from each patient taking part in the study. Participants confirmed their willingness to participate in the study and their permission for researchers to access their medical records.

### Data collection

#### Colorectal cancer cohort

The CRC cohort was recruited from an outpatient hospital clinic in the VIII Health Care Area of Asturias over four years (2008-2012). A total of 267 CRC patients were finally enrolled. Most of these were referred by primary care-givers to the Gastroenterology Department for diagnostic purposes and proper management, and from there, some of them were later referred to outpatient clinics from their referral hospital, to evaluate the need for surgery or other types of treatment.

A database was set up containing information from all patients about their general demographic characteristics, medical history, and the results of physical examination, laboratory tests, colon endoscopy, colorectal biopsies, and various radiological tests. Tumor stage and location were classified following the Union for International Cancer Control (UICC) recommendations [18]. When required, the corresponding author provided genetic counselling to the AAT-deficient patients and their families.

#### Control cohort

327 volunteer healthy unrelated people (HUP) from the VIII Health Care Area were recruited by simple random sampling. To do this, people were selected from the region’s municipal census records through the use of random numbers generated by the R-Sigma statistical program. To standardize the two series, only people between 40 and 90 years, 60-70% of them male, were chosen for possible inclusion. Explanatory letters were sent to them and their cooperation with the study invited. We also contacted the primary care services and health area municipalities to encourage participation by the potential subjects.

A general clinico-epidemiological questionnaire was completed by each suitable volunteer. Only healthy people were allowed to participate in the study, those with serious diseases being rejected. Blood samples were most commonly obtained at the Valle del Nalón Hospital laboratory, but some were collected at the health centres in the area, according to the participants’ preferences. Besides the measurements related to the subject of the study (AAT serum concentrations and Pi phenotypes), routine haematological and biochemical analyses were performed, and 5-8 aliquots of serum from each person were reserved to check results when these indicated that it might be appropriate to carry out other studies. Individual letters were sent to participants with normal analytical results, and Z allele carriers were contacted in an effort to persuade them to take part in studies, long-term follow-up and family screening.

### Serum AAT and Pi system phenotypes

Serum AAT levels were determined in the reference laboratory of the Instituto Nacional de Silicosis (Oviedo) by nephelometry, with an Array™ Protein System autoanalyzer (Beckman Instruments, Brea, California, USA). The normal range of values in our laboratory is 100-220 mg/dL.

Phenotypes were characterized in the Instituto Nacional de Silicosis by isoelectric focusing (IEF) with a HYDRAGEL 18 A1AT isofocusing kit, designed for the qualitative detection and identification of the different AAT phenotypes in the electrophoretic patterns of human sera. The procedure involves IEF in agarose gel performed in the automatic HYDRASYST system, followed by immune-fixation with AAT antiserum (SEBIA Hispania S.A., Barcelona, Spain).

#### Pi allelic frequency and phenotypic prevalence

Gene frequency is defined as the frequency of all genes of a particular type, whether occurring in homozygotes or heterozygotes. The total number of alleles is twice the number of subjects. Therefore, the gene frequency was obtained by adding the number of S or Z alleles, and expressing this total as a fraction of the total number of Pi alleles in the population (alleles per 1,000 genes of all Pi types).

The prevalence of each phenotype was calculated assuming the population to be in Hardy-Weinberg equilibrium: p^2^ + 2pq + q^2^ = 1 (where p = proportion of the Z allele, and q = proportion of the S allele). This formula was used to estimate the prevalence of Z homozygotes and the SZ heterozygotes [19].

#### Precision factor score (PFS) of statistical reliability for each cohort

To assess the statistical reliability of the results, a coefficient of variation (CV) for Pi*S and Pi*Z frequencies in each cohort was calculated. This CV is a measure of the precision of results from each cohort in terms of the dispersion of the data around the mean. Its value depends on the number of alleles studied and on the frequencies of Pi*S and Pi*Z actually found. The precision is inversely proportional to the CV. Numerical precision factor scores (PFS) for assessing the statistical quality and precision of each cohort were generated as follows, from both S and Z CVs:

$$Z_{\mathrm{CV}}=\frac{100\times \left(Z_{\mathrm{ul}}-Z_{\mathrm{ll}}\right)}{4\times Z_{\mathrm{fr}}}$$

and

$$S_{\mathrm{CV}}=\frac{100\times \left(S_{\mathrm{ul}}-S_{\mathrm{ll}}\right)}{4\times S_{\mathrm{fr}}}$$

The mean CV value was calculated as:

$$\bar{\mathrm{CV}}=\frac{Z_{\mathrm{CV}}+S_{\mathrm{CV}}}{2}$$

and the numerical PFS was calculated as follows:

$$\mathrm{PFS}=500\times \frac{1}{\bar{\mathrm{CV}}}\;$$

(where S_ul_ = 95% CI upper limit of S; S_ll_ = 95% CI lower limit of S; Z_ul_ = Z 95% CI upper limit of Z; Z_ll_ = Z 95% CI calculated lower limit; S_fr_ = frequency of S; Z_fr_ = frequency of Z. The factor of 500 ensures a PFS value scaled from 0 to 12). These statistical calculations provide estimates of the mean, median, standard deviation and the range of the PFS in each cohort. An appropriate value of PFS for the Asturias population should be greater than 8 [20].

### Statistical analysis

Descriptive statistics were used to tabulate the primary cohort database. Quantitative variables were expressed as the mean and standard deviation (SD). The normality of the distributions of quantitative variables was tested by the Kolmogorov-Smirnov test. Serum concentrations were compared using Student’s unpaired samples t-test. A value of p < 0.05 was considered to be statistically significant.

## Results

The CRC cohort consisted of 267 subjects, 63% of whom were males, with a mean age of 72 years (range: 44-90 years). The control cohort comprised 327 subjects, 67% of whom were males, with a mean age of 70 years (range: 42-89 years). No significant differences in demographic features were found (Table 1).

**Table 1:** Demographic features found in the general population (control cohort) and in the colorectal cancer cohort

General population, AAT-Pi* genotypes							Colorectal cancer, AAT-Pi* genotypes						**P**
	**Total**	**MM**	**MS**	**MZ**	**SS**	**SZ**	**Total**	**MM**	**MS**	**MZ**	**SS**	**SZ**	
**Genotypes, n (%)**	327 (100)	256 (78.3)	60 (18.3)	9 (0.03)	1 (.003)	1 (.003)	267 (100)	207 (77.5)	49 (18.4)	10 (0.04)	0 (0)	1 (0.004)	0.985
**Males, n (%)**	219 (67)	171 (67)	40 (67)	7 (78)	0 (0)	1 (100)	169 (63)	131 (63)	32 (65)	6 (60)	-	-	0.712
**Age, *years *** **[SD]**	70.0 [7.5]	69.8 [7.4]	70.5 [7.9]	73.2 [6.4]	65.0 -	80.0 -	72 [9.6]	72.5 [9.8]	72.8 [9.2]	70.2 [8.1]	-	81 [NA]	0.713
**Familial CRC, n (%)**	13 (3.9)	12 (5)	1 (1.7)	0 (0)	0 (0)	0 (0)	11 (4)	10 (5)	1 (2)	0 (0)	-	0 (0)	0.590
Alcohol abuse, n (%)*	66 (20)	60 (23)	5 (8)	1 (11)	0 (0)	0 (0)	34 (13)	27 (13)	5 (10)	2 (20)	-	0 (0)	0.706
TOB n (%)**	88 (27)	77 (30)	8 (13)	2 (22)	0 (0)	1 (100)	103 (39)	84 (41)	16 (33)	3 (30)	-	0 (0)	0.640
**NSAIDS n (%)**	4 (1)	3 (1)	0 (0)	1 (11)	0 (0)	0 (0)	34 (12)	25 (12)	9 (18)	0 (0)	-	0 (0)	0.345
OW, n (%)***	10 (3)	8 (3)	1 (2)	1 (11)	0 (0)	0 (0)	19 (7)	17 (8)	1 (2)	1 (10)	-	0 (0)	0.384
**CWPC, n (%)**	2 (1)	0 (0)	0 (0)	1 (11)	0 (0)	1 (100)	25 (9)	22 (10)	2 (4)	1 (10)	-	0 (0)	0.421
**ATH, n (%)**	124 (38)	113 (44)	10 (17)	1 (11)	0 (0)	0 (0)	112 (42)	90 (44)	18 (37)	4 (40)	-	0 (0)	0.799
**DL, n (%)**	40 (12)	36 (14)	4 (7)	0 (0)	0 (0)	0 (0)	56 (21)	43 (21)	9 (18)	3 (30)	-	1 (100)	0.491
**DM n (%)**	18 (5)	17 (7)	1 (2)	0 (0)	0 (0)	0 (0)	50 (19)	42 (20)	6 (12)	2 (20)	-	0 (0)	0.519
CLD, n (%)****	0 (0)	-	-	-	-	-	17 (6)	13 (6)	3 (6)	1 (10)	-	0 (0)	1.000

Pi* protease inhibitor. *AAT* alpha-1 antitrypsin, *n* number. * > 40 g ethanol consumed/day. **Current smokers or ex-smokers of >15 packs/year. *OW* overweight, ***BMI 30 kg/m^2^. *CLD* chronic liver disease, ****Mostly, chronic hepatitis and/or liver cirrhosis. *CWPC* coal workers’ pneumoconiosis. *DM* diabetes mellitus. *ATH* arterial hypertension. *NSAIDS* chronic intake of non-steroidal anti-inflammatory drugs. *TOB* tobacco abuse. *DL* Dyslipidemia. No significant differences were found in any of the parameters.

Sample sizes, PFS values, number and types of AAT alleles, along with Pi*S and Pi*Z gene frequencies, and prevalences calculated assuming the Hardy-Weinberg equilibrium for the two cohorts are shown. The frequency of the severe deficiency allele Pi*Z and the estimated prevalence of MZ, SZ and ZZ were numerically higher in CRC patients than in HUP subjects, although the differences were not statistically significant (Table 2).

**Table 2:** PFS, allele type, mean gene frequency and prevalence in both cohorts

**Sample (n)**	**PFS**	**Total alleles**	**Allele types (n)**			Pi*S and Pi*Z mean gene frequencyin x 1,000 [95% CI]		**Hardy-Weinberg calculated prevalence [1/x]**					
			**M**	**S**	**Z**	Pi*S	Pi*Z	**MM**	**MS**	**MZ**	**SS**	**SZ**	**ZZ**
**General population (327)**	5.4	654	581	63	10	96 [75-122]	15 [8-29]	1.3	6	37	108	339	4,267
**Colorectal cancer (267)**	5.4	534	473	50	11	94 [71-122]	21 [11-38]	1.3	6	27	114	259	2,357
*P-value*						0.954	0.639						

*N* number, *PFS* precision factor score (scale 0-12), *CI* confidence interval, Pi* protease inhibitor. No significant differences were found in any of the comparisons.

We found significant differences in AAT serum concentrations between the AAT phenotypes of the studied cohorts, with notably higher values in CRC patients than in HUP subjects (p < 0.001) (Table 3).

**Table 3:** Mean serum concentration of alpha-1 antitrypsin in the phenotypes in both cohorts

**Sample**	**Mean serum concentration of alpha-1 antitrypsin (mg/dl)**				
	**MM**	**MS**	**MZ**	**SS**	**SZ**
**General population**					
**N**	256	60	9	1	1
**Mean**	143.8	122.9	90.2	107.5	71.2
**SD**	(20.5)	(30.6)	(10.7)	-	-
**Range**	[88-235]	[75-202]	[75-107]	[107.5]	[71.2]
**Colorectal cancer**					
**N**	206	49	10	0	1
**Mean**	208	167.7	139.2	-	124
**SD**	(60)	(40.8)	(31.3)	-	-
**Range**	[105-459]	[93-278]	[84-183]	-	[124]
**P**	0.0001	0.0001	0.001	NA	NA

*N* number, *SD* standard deviation, *NA* not applicable. Significant differences found between MM, MS and MZ.

All cases included in our study were carriers of adenocarcinomas. The anatomical location of these cancers, their TNM stage, the treatment given to each patient, as well as any deaths and their causes are summarized in Table 4. CRC patients with the MZ genotype tended to have more advanced tumors (i.e, Stage III) than did those of the MM normal genotype (50% *vs.* 34%). In addition, 60% of MZ patients received postoperative chemotherapy, whereas only 30% of MM patients did (p = 0.058). 30% of MZ patients compared with 16% of the MM subgroup died from causes directly related to the CRC, the difference not being statistically significant. The analysis of the remaining descriptive data falls outside the scope of this study, and is presented for information purposes only.

**Table 4:** Colorectal cancer anatomical location, TNM stage, treatment and follow-up, in the different AAT-Pi phenotypes

		AAT Pi* genotype				
	**Total n (%)**	Pi*MM n (%)	Pi*MS n (%)	Pi*MZ n (%)	Pi*SZ n (%)	**P**
**Location**						0.822
Ascending colon	50 (19)	40 (19)	7 (14)	3 (30)	0 (0)	
Transverse colon	22 (8)	18 (9)	3 (6)	1 (10)	0 (0)	
Descending colon	30 (11)	24 (12)	4 (8)	2 (20)	0 (0)	
Sigmoid colon	91 (34)	68 (33)	21 (43)	1 (10)	1 (100)	
Rectum	74 (28)	57 (27)	14 (29)	3 (30)	0 (0)	
**TNM stage**						0.409
I	33 (12)	25 (12)	6 (12)	2 (20)	0 (0)	
II	124 (47)	92 (44)	29 (59)	2 (20)	1 (100)	
III	85 (32)	71 (34)	9 (19)	5 (50)	0 (0)	
IV	25 (9)	19 (10)	5 (10)	1 (10)	0 (0)	
**Treatment**						
Surgery	255 (96)	197 (95)	47 (96)	10 (100)	1 (100)	1.000
Chemotherapy	79 (30)	63 (30)	10 (20)	6 (60)	0 (0)	0.058
Radiation	43 (16)	31 (15)	9 (18)	3 (30)	0 (0)	0.559
Chemoradiotherapy	20 (8)	16 (8)	3 (6)	1 (10)	0 (0)	0.921
Palliative care	12 (4)	10 (5)	2 (4)	0 (0)	0 (0)	NA
**Deaths**	62 (23)	45 (22)	14 (29)	3 (30)	0 (0)	NA
CRC-related	46 (17)	33 (16)	10 (20)	3 (30)	0 (0)	NA
Other causes of death:	16 (6)	12 (6)	4 (8)	0 (0)	0 (0)	NA
*Acute myocardial infarction*	3	2	1			NA
*Congestive heart failure*	1	1				NA
*Gastrointestinal bleeding*	2	2				NA
*Postsurgical sepsis*	3	3				NA
*Post-chemotherapy sepsis*	2		2			NA
*Diffuse non-Hodgkin’s lymphoma*	1	1				NA
*Pulmonary thromboembolism*	2	2				NA
*Brain stroke*	1	1				NA
*Hepatic failure liver cirrhosis-related*	1		1			NA

*TNM* tumor stage classification according to primary tumor invasion proof (T), regional lymph nodes involved (N), and presence of distant metastasis (M), *NA* not applicable. *CRC* colorectal cancer. No significant differences were found in any of the comparisons.

Finally, we have not found significant differences (p = 0.502) from the comparison of the mean value in AAT serum concentrations of the whole CRC group and each CRC stage (I- IV) (Table 5).

**Table 5:** **Comparison of serum concentrations of AAT in the group of patients with colorectal cancer (total and classified by TNM stages) *vs. *** **controls**

	**GP controls**	**CRC**	**TNM stage of CRC cases**			
	**Total**	**Total**	**I**	**II**	**III**	**IV**
**Number of subjects (%)**	327 (100)	267 (100)	33 (12)	124 (47)	85 (32)	25 (9)
**Mean serum AAT (SD)**	138.5 (25.3)	198.1 (58.0)	197.9 (63.0)	194.3 (60.1)	198.9 (55.7)	214.5 (59.3)
	P-value A*: <0.001	P-value B**: 0.502				

CRC colorectal carcinoma. *GP* general population. Serum concentration values are expressed in mg/dL. *SD* standard deviation.

P-value A*: from the comparison of mean values of serum AAT in the control and CRC groups, and between the mean AAT in the control group and the partial AAT value for each TNM subgroup.

P-value B**: from the comparison of the mean value of the whole CRC group and each CRC stage (I- IV).

## Discussion

The only statistically significant finding of the present study was the markedly higher AAT serum concentrations in CRC patients than in healthy controls, regardless of whether their Pi phenotype was normal (Pi*MM) or deficient (Pi*MS, Pi*MZ or Pi*SZ).

The presence of high serum levels of AAT in patients with CRC was reported more than 35 years ago, and has even been linked to distant metastases [21]. Subsequently, other authors have found that serum AAT levels are associated with the clinical stage of the disease [22,23]. In these pioneering studies, the correlation of serum CEA and serum AAT with the stage of disease were of a very similar level of statistical significance (p = 0.004 and 0.003, respectively). Coinciding with these preliminary results, a more recent study confirmed that serum levels of AAT are higher in CRC subjects than in controls, and that these high levels of serum AAT are directly correlated with the stage of CRC, making it a useful marker for distinguishing between early and advanced stages of this malignancy [24]. However, given the necessarily strict criteria, we cannot yet be certain whether this biomarker is also altered in patients with other inflammatory or neoplastic diseases.

Apart from CRC, various authors have found significantly elevated AAT serum levels in subjects with a range of cancers, including lung [25-30], liver [31,32], pancreas [33], prostate [34], cervix [35], ovary [36,37], breast [38], Hodgkin’s lymphoma [39], larynx and other head and neck carcinomas [40,41]. The data provided by these studies taken together suggest that the presence of elevated serum levels of AAT in patients with any of these types of carcinomas is related to an invasive growth of these tumors. However, the low statistical power of the analyses that is the consequence of the small sample sizes means that the true value of this biomarker in the diagnosis and staging of cancers remains to be established.

On the other hand, AAT has been detected in histological sections of paraffin-embedded biopsy specimens obtained by endoscopy or surgically resected CRC samples, with a markedly higher incidence in advanced than in early carcinomas. These findings suggest a local production of AAT by CRC cells that tends to be associated with a more aggressive tumor behavior, more intense local growth and an increased tendency to metastasize to distant organs [42]. However, AAT overexpression in cancer tissues is not an exclusive feature of CRC, since it has also been found in other types of cancers in different organs, including lung carcinomas [43], hepatocellular carcinomas [44], adenocarcinomas of the stomach [45,46], myeloid leukemia cells [47,48], brain tumors [49], carcinoid tumors, malignant melanomas, and schwannomas [50]. *In vitro* production of AAT by tumor cells themselves also occurs in a variety of adenocarcinoma, sarcoma, glioblastoma and chordoma cell lines [51,52]. Based on the results of these studies, the presence of AAT in tumors has typically been ascribed to its production by the tumor cells themselves, and patients with AAT expression in their tumors have been thought to have a worse prognosis than those without AAT expression.

However, two recently published studies have provided results that call into question these previously accepted concepts. Firstly, a study of tissue expression of AAT in a 372-dot tissue array, and its concentrations in sera of patients with CRC, using a methylation isotope-labeling-assisted gel-enhanced liquid chromatography-mass spectrometry strategy, found that CRC specimens expressed less AAT in both tissues and serum than did normal counterparts [53]. This surprising result was supported by a subsequent study of gastric cancer tissues and adjacent normal tissues obtained from surgery, using two-dimensional differential gel electrophoresis, validating protein expression by western blot and IHC, which found AAT to be significantly downregulated in gastric cancer patients [54]. *In vitro* comparative analysis of the human tumors and normal tissues revealed an association between reduced local AAT expression and more aggressive tumor growth [55].

Nevertheless, the role that AAT may play in tumor invasiveness is currently unknown. It has been suggested that since neutrophil elastase is present in colon carcinoma tissues, and its level is very similar to the degree of tissue infiltration by neutrophils, it is possible that an excess of free elastase promotes a favorable host environment for carcinogenesis [56]. Other authors have linked carcinogenesis to AAT degradation by matrix metalloproteinases activated by neutrophil elastase, cathepsin G, and proteinase-3 [57], ultimately resulting in the production of COOH-terminal fragments, which boosts tumor growth *in vivo*[58].

In addition to the markedly elevated AAT serum levels found in CRC patients compared with controls, other results of our study merit discussion, even though the small sample size and the marked deviation from the mean of some values meant that these differences between cases and controls were not statistically significant. Briefly, these findings were as follows: (1) CRC cases in advanced stages (III and IV) had higher AAT serum concentrations than those in early stages (I and II); (2) the gene frequency of the severe deficiency Pi*Z allele, and the prevalence of the Pi*MZ, Pi*SZ and Pi*ZZ deficiency phenotypes were higher in CRC patients than in controls; and (3) CRC patients with the Pi*MZ genotype tended to develop more locally advanced tumors, had a greater need for postoperative chemotherapy, and had a greater rate of mortality from causes directly related to the CRC than did subjects with the MM genotype.

Nonetheless, our results cast some doubt on the accuracy of the present study, because it might be biased by the small size of the samples studied, as suggested by the low PFS displayed by the two cohorts (both 5.4 points). This low value would require both samples to be approximately doubled in size to improve it sufficiently.

There is wide-ranging evidence about the relationship between AAT deficiency and the development of various types of malignancy, including CRC. The level of evidence, in terms of evidence-based medicine, is high with respect to the risk of subjects with Pi*ZZ genotype developing hepatocellular carcinomas, which reaches the very high percentage of 28% [1,4,5]. Regarding lung cancer, two studies found Pi*MS and Pi*MZ heterozygote individuals to be at increased risk of developing bronchial carcinomas, particularly of the squamous and bronchoalveolar cell types, independent of smoking habit and presence of COPD [6,7]. The mechanism involved in lung carcinogenesis would be an excess of neutrophil elastase that is not neutralized by AAT and that stimulates development, invasion and metastasis. This same mechanism would probably be shared by all other types of cancers, including CRC [8,9]. There is also some evidence of a relationship between AAT deficiency and the development of neoplasms of the urinary bladder and gallbladder, and malignant lymphomas [10-12].

Colorectal cancer, a leading cause of cancer deaths worldwide, has also been associated with AAT deficiency [13,14]. It is known that both normal and cancer intestinal cells secrete AAT [56,59] to neutralize elastase, which is present in high concentrations in colon carcinoma cells, in an attempt to maintain the protease-antiprotease balance. This prevents the activation of procathepsin B and proprotein convertase, and reduces the production of TNF-α and IL-1a, which prevents liver metastases [60-62]. However, the only two clinico-epidemiological studies carried out to date produced conflicting results [13,14]. The first study, of patients with CRC and a microsatellite instability genotype, found a significantly higher prevalence of AAT deficiency alleles in CRC subjects than in the general population (21.6% *vs.* 9.4%), and that smokers with AAT deficiency had a 20-times greater risk than expected of developing high microsatellite instability compared with smokers without AAT deficiency [13]. Conversely, a more recent case-control study confirmed the link between smoking history and the high degree of microsatellite instability, but no difference in AAT deficiency frequency between cases and controls, irrespective of their microsatellite unstable subtype [14].

## Conclusions

Our study found that patients with CRC have much higher serum AAT concentrations than healthy controls, regardless of the genotypes of the subjects. This finding is consistent with most published classic studies, but is unlike others published recently. Its meaning is therefore uncertain, and its potential role in the diagnosis and staging of CRC remains to be established. Further studies are needed in other diseases and other gastrointestinal tumors to determine the sensitivity and specificity of this biomarker.

On the other hand, based on our findings, our initial hypothesis that AAT deficiency is involved in the development and progression of CRC could neither be confirmed nor ruled out, since a trend towards more severe AAT deficiency with more advanced tumor stage was observed. Not enough Z alleles were analyzed in our study for statistical significance to be reached for an effect size of the observed magnitude. Similar studies but of greater statistical power are therefore required to settle this matter.

## Abbreviations

AAT: Alpha-1 antitrypsin; α1-Pi: Alpha-1 proteinase inhibitor; ≤COPD: Chronic obstructive pulmonary disease; CRC: Colorectal cancer; CV: Coefficient of variation; HUP: Healthy unrelated people; IEF: Isoelectric focusing; IL: Interleukin; PAR: Proteinase-activated receptor; PFS: Precision factor score; Pi: Protease inhibitor.; Pi*M: Protein M-dependent protease inhibitor gene, resulting in a normal genotype; Pi*MM: Genotype composed of two Pi*M alleles; Pi*MS: Genotype composed of one Pi*M allele and one Pi*S allele, resulting in a slightly deficient genotype; Pi*MZ: Genotype composed of one Pi*M allele and one Pi*Z allele, resulting in a moderately deficient genotype; Pi*S: Protein S-dependent protease inhibitor gene, resulting in a moderately deficient genotype; Pi*SS: Genotype composed of two Pi*S alleles, resulting in a moderately deficient genotype; Pi*SZ: Genotype composed of one Pi*S allele and other Pi*Z allele, resulting in a severely deficient genotype; Pi*Z: Protein Z-dependent protease inhibitor gene, resulting in a severely deficient genotype; Pi*ZZ: Genotype composed of two Pi*Z alleles, resulting in a severely deficient genotype; SD: Standard deviation; SERPINA1: Serine protease inhibitor, group A, member 1; UICC: Union for International Cancer Control.

## Competing interests

The authors declare that they have no competing interests.

## Authors’ contributions

Dr. IB, designed the study, helped acquire the control cohort data, and bibliography collection, and wrote the manuscript. Prof. LR, contributed to the revision of the statistical analysis, and approved the final version of the manuscript. Dr. MM, developed the laboratory analysis and results. Dr. SP-H, contributed to the conception of the study, the acquisition of the case cohort data, and the interpretation of data comparisons; he coordinated the study, and wrote the first draft of the manuscript. All authors have read and approved the final manuscript.

## Pre-publication history

The pre-publication history for this paper can be accessed here:

http://www.biomedcentral.com/1471-2407/14/355/prepub
